# Platelet-to-albumin ratio: a risk factor associated with technique failure and mortality in peritoneal dialysis patients 

**DOI:** 10.1080/0886022X.2021.1977319

**Published:** 2021-09-30

**Authors:** Yuqi Yang, Jing Yuan, Lu Liu, Shuwen Qie, Li Yang, Zha Yan

**Affiliations:** a Department of Nephrology, Guizhou Provincial People’s Hospital, Guiyang, China; b NHC Key Laboratory of Pulmonary Immunologic Disease, Guizhou Provincial People’s Hospital, Guiyang, China

**Keywords:** Platelet-to-albumin ratio, technique failure, mortality, peritoneal dialysis

## Abstract

**Background:**

Peritoneal dialysis (PD) patients have a high incidence of poor clinical outcomes, which is related to the inflammatory and nutritional status of this population. Platelet-to-albumin ratio (PAR), recently identified as a useful biomarker to monitor inflammation and nutrition, can predict a poor prognosis in various diseases. The aim of this study was to investigate the association between PAR and technique failure and mortality in PD patients.

**Methods:**

This single-center retrospective study enrolled 405 PD patients from 1 January 2011 to 31 December 2019 and collected complete demographic characteristics, clinical laboratory baseline data. The outcomes were technique failure and mortality. The associations between PAR and technique failure, death were analyzed by Cox proportional hazard models and competing risk regression models with kidney transplantation as a competing event. The areas under the curve (AUC) of receiver-operating characteristic analysis were used to determine the predictive values of PAR for technique failure and mortality.

**Results:**

During a median follow-up period of 24.0 (range, 4.0–91.0) months, 139 (34.3%) PD patients experienced technique failure, 61 (15.1%) PD patients died. The patients with higher PAR levels had increased risk of technique failure and mortality. After adjustment for confounding factors, we found that high PAR levels were risk factor for both technique failure (subdistribution hazard ratio [*SHR*] 1.775; *95%CI*, 1.157–2.720; *p* = 0.033] and mortality [*SHR* 3.710; *95%CI*, 1.870–7.360; *p* < 0.001]. The predictive ability of PAR was superior to platelet and albumin based on AUC calculations for technique failure and mortality.

**Conclusions:**

PAR was a risk factor associated with technique failure and mortality in PD patients.

## Introduction

Peritoneal dialysis (PD) is an established treatment modality of kidney replacement therapy for patients with kidney failure increasing acceptance worldwide [1,2]. Despite PD technology has significantly advanced, high technique failure rates, high hospitalization rates, and high mortality rates remain tough challenges [3–5]. Therefore, more efforts should be made to identify the risk of PD technique failure and mortality, to act on modifiable risk factors, offer enhanced preventative strategies to vulnerable patients.

Accumulating evidence has demonstrated that inflammation is an important risk factor of poor prognosis for PD patients. Beyond the antithrombotic effects, platelets can also trigger and exacerbate inflammation through interaction with a variety of immune cells and secretion of proinflammatory cytokines [6]. Higher platelet counts have been proven to be associated with higher risk of cardiovascular-associated mortality in PD patients [7]. Moreover, recent studies have demonstrated that platelet-to-lymphocyte ratio (PLR), a platelet-associated inflammatory parameter, is a risk factor of cardiovascular-associated events and mortality in PD patients [8,9]. Malnutrition is another important prognostic marker for accessing poor outcomes in PD patients [10]. Low serum albumin level, generally recognized as a crucial indicator for nutritional status, is associated with an increased risk of technique failure and mortality in PD patients [11,12]. Previous studies have suggested that inflammation drives the development of malnutrition, which may in turn amplify systemic inflammatory responses, leading to a vicious cycle [13]. Therefore, a comprehensive assessment of inflammatory and nutritional status will provide more assistance to recognize the risk factors of technique failure in PD patients. More recently, platelet to albumin ratio (PAR), a composite indicator of inflammatory and nutritional status, has been proven as a useful and potential prognostic biomarker in various cancer, including cholangiocarcinoma [14] and nonsmall-cell lung cancer [15]. However, to date, few studies have investigated the association between PAR and clinical outcomes in PD patients. We aimed to evaluate the PAR value in predicting technique failure and mortality in PD patients.

## Materials and methods

### Study design and participants

The patients commencing PD as the first kidney replacement therapy at the Department of Nephrology, Guizhou Provincial People’s Hospital between 1 January 2011 and 31 December 2019 were recruited. The inclusion criteria for patients were as follows: (1) over 18 years old; (2) regular PD for more than 3 months. Exclusion criteria were as follows: (1) received hemodialysis or kidney transplantation prior to PD; (2) combined with recent active infection, malignancies, liver diseases, hematological diseases or active autoimmune diseases; (3) incomplete data of platelet counts or albumin levels. The study was performed according to the ethic requirements of Guizhou Provincial People’s Hospital Human Research Ethics Committee ([2020]208) and complied with the principles of the Declaration of Helsinki for medical research.

### Data collection

Baseline characteristics at the initiation of PD therapy, including age, gender, causes of kidney failure, comorbidities including a history of cardiovascular disease (CVD), hypertension, diabetes, body mass index (BMI), mean arterial pressure (MAP) levels were obtained from medical records. A history of CVD was defined as a patient who had one or more of the following CVD: angina, myocardial infarction, heart failure, angioplasty, coronary artery bypass or stroke. Hypertensive patients were those who had at least two separate blood pressure measurements above 140/90 mmHg and/or those who used antihypertensive drugs currently or previously. Diabetic patients were those who met the clinical diagnostic criteria for diabetes mellitus and/or those who currently or previously used insulin or oral hypoglycemic agents.

The laboratory parameters within three months after initiation of PD were collected. They included leukocytes, neutrophils, lymphocytes, platelets and hemoglobin levels; serum albumin, creatinine and uric acid; serum sodium, potassium, chlorine, calcium, phosphorus, alkaline phosphatase, intact parathyroid hormone; serum triglyceride, cholesterol, low-density lipoprotein cholesterol (LDL-C), high-density lipoprotein cholesterol (HDL-C); hypersensitive C-reactive protein (hs-CRP). All laboratory data were measured using automated systems and standard methods. PAR was calculated by dividing absolute platelet counts by serum albumin levels. Neutrophil-to-lymphocyte ratio (NLR) and PLR were calculated by dividing absolute neutrophil counts and platelet counts, respectively, by absolute lymphocyte counts. Body mass index (BMI) was obtained as weight/height^2^ (kg/m^2^). We estimated residual kidney function (RKF) by calculating the residual glomerular filtration rate (GFR) with the Chronic Kidney Disease Epidemiology Collaboration (CKD-EPI) equation [16].

### Study outcomes

The primary outcome was technique failure, the second outcome was all-cause mortality. Technique failure was defined as transfer to hemodialysis therapy for more than 30 days or death on PD therapy or within 30 days of transfer to hemodialysis therapy [17]. All the patients were followed up until death, transfer to hemodialysis, kidney transplantation, transfer to other centers, loss to follow-up or the end of follow-up on 31 December 2020.

### Statistical analysis

The study population was subdivided into three groups according to the PAR. Continuous variables were expressed by mean values with standard deviation (SD) if normally distributed or median and interquartile range (IQR) if not normally distributed and categorical variables by frequencies and percentages. Differences among the PAR groups were compared using the Kruskal–Wallis tests for continuous variables and the Chi-squared test for categorical variables. The correlations between PAR and other clinical data were analyzed with correlation analysis.

Kaplan–Meier method was used to estimate and plot survival curves of technique survival and mortality. The differences were assessed using the log-rank test. Factors associated with technique failure and mortality were examined by Cox proportional hazards and competing risk analysis. A univariate analysis model was used to investigate the relationship between each independent variable, and multivariate analysis model was used to determine the independent variables that continued to have associations with outcomes after including significant variables in the univariate analysis. We also used competing risk regression model using the method described by Fine and Gray, and kidney transplantation was considered as a competing event. The covariates for Cox proportional hazards models and competing risk analysis were the same. Results were expressed as hazard ratio (HR) and subdistribution hazard ratios (SHR) with 95% confidence intervals (95%CI). The PAR values with the first triplicate were selected as the Reference. The receiver operating characteristic (ROC) curves were used to analyze the predictive power of PAR, platelet and albumin for technique failure and mortality. A two-tailed *p*-value < 0.05 was considered to indicate a statistically significant difference. SPSS version 23.0 (IBM Corp., Armonk, NY, USA) was used for the data input and statistical analysis. R statistical software (R Foundation for Statistical Computing, Vienna, Austria, http://www.R-project.org/) was used for competing risk analysis.

## Results

### Demographic and clinical characteristics of the study population

**Figure 1.**
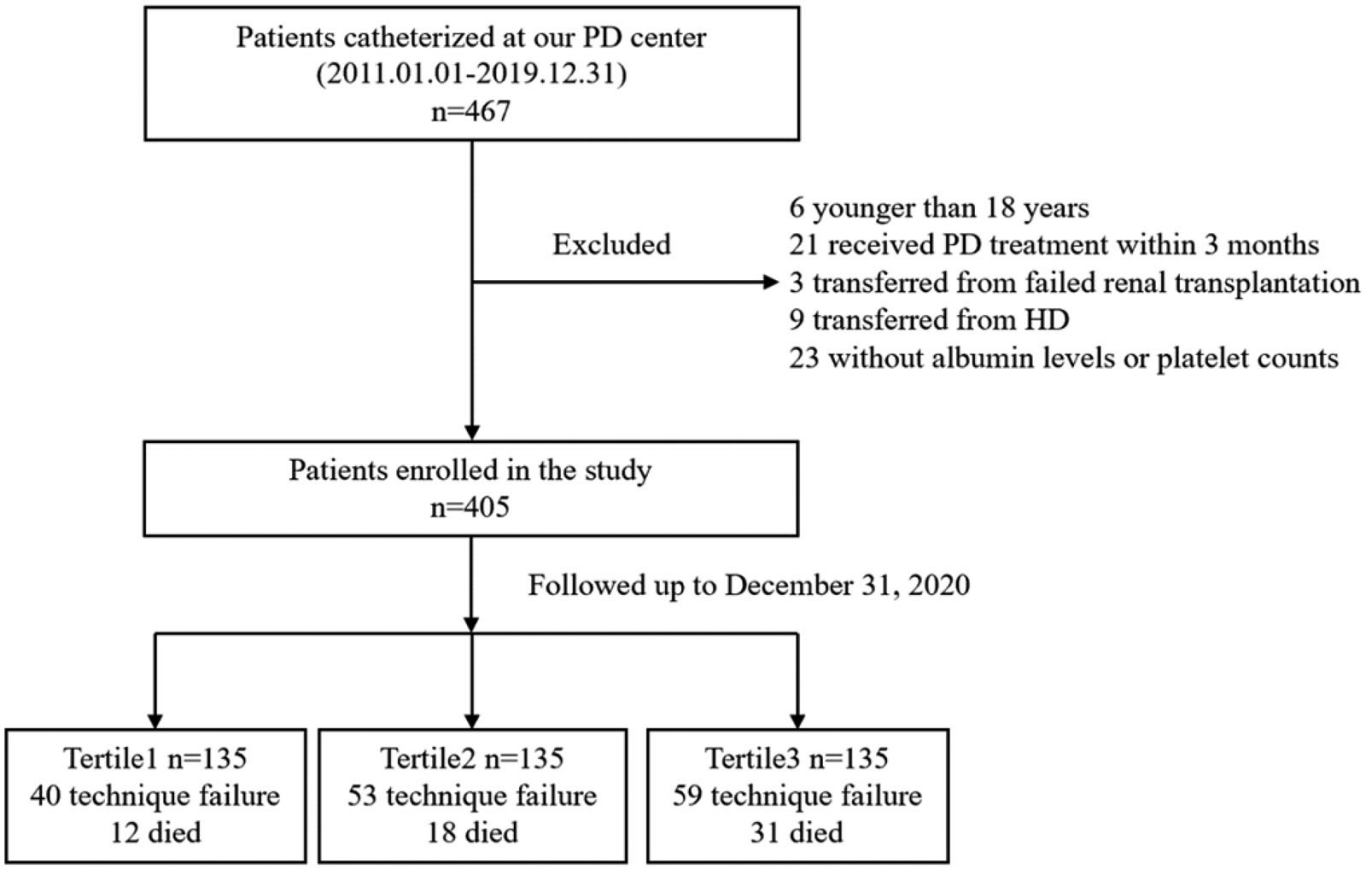
Flow chart of the study. PD: peritoneal dialysis; HD: hemodialysis.

There were 467 incident patients who had commenced PD between1 January 2011 to 31 December 2019 in our center. Of these, six patients were younger than 18 years, 21 had received PD treatment for less than 3 months, 23 had missing data on basic albumin levels or platelet counts, nine transferred from HD and three experienced failed kidney transplantation. Finally, a total of 405 patients were enrolled in this study (Figure 1). There were 209 males (51.6%) and 196 females (48.4%), with an average age of 39.2 ± 12.8 years old. The etiology of kidney failure in patients were glomerulonephritis in (71.4%), diabetic nephropathy in (8.9%), hypertensive kidney disease in (11.1%) and other reasons in (8.6%). They were divided into three groups according to the PAR levels: tertile 1 (PAR < 4.51, *n* = 135); tertile 2 (PAR4.51-6.27, *n* = 135); tertile 3 (PAR > 6.27, *n* = 135). Table 1 summarized the demographic characteristics and hematologic parameters of the PAR groups. Compared other two groups, the patients in tertile 3 had higher levels of leukocyte, neutrophil, lymphocyte, platelet counts and serum cholesterol, LDL-C and lower serum levels of sodium, potassium and albumin (*p* < 0.05 for each). Importantly, the inflammatory markers, hs-CRP, NLR and PLR levels were highest in the tertile 3 (*p* < 0.05 for each). There were no significant differences among groups in sex, age, BMI, MAP, cause of kidney failure, comorbidity, RKF, levels of hemoglobin, calcium, phosphorus, parathyroid hormone, alkaline phosphatase, uric acid, triglyceride and HDL-C (*p* > 0.05).

**Table 1.** Baseline characteristics of PD patients stratified by the PAR.

Variables	Total (*n* = 405)	PAR			*p* Value
		Tertile 1 (<4.51) (*n* = 135)	Tertile 2 (4.51-6.27) (*n* = 135)	Tertile 3 (>6.27) (*n* = 135)	
PAR	5.3 (4.1,7.0)	3.6 (3.1,4.1)	5.3 (4.9,5.9)	8.0 (6.9,9.0)	<0.001
Age (years)	39.2 ± 12.8	38.9 ± 13.4	38.7 ± 12.5	40.0 ± 12.6	0.659
Men (n, %)	209 (50.4%)	76 (56.3%)	75 (55.6%)	58 (43.0%)	0.048
BMI (kg/m^2^)	21.5 ± 3.7	21.2 ± 3.6	22.0 ± 3.9	21.2 ± 3.4	0.187
Primary cause of kidney failure					
Glomerulonephritis	289 (71.4%)	99 (73.3%)	92 (68.1%)	98 (72.6%)	0.595
Diabetic kidney disease	36 (8.9%)	8 (5.9%)	11 (8.1%)	17 (12.6%)	0.146
Hypertensive kidney disease	45 (11.1%)	15 (11.1%)	17 (12.6%)	13 (9.6%)	0.741
Others	35 (8.6%)	13 (9.6%)	15 (11.1%)	7 (5.2%)	0.197
Comorbidity					
Hypertension	357 (88.1%)	119 (88.1%)	121 (89.6%)	117 (86.7%)	0.824
Diabetes mellitus	44 (10.9%)	10 (7.4%)	14 (10.4%)	20 (14.8%)	0.260
Cardiovascular disease	66 (16.3%)	19 (14.1%)	22 (16.3%)	25 (18.5%)	0.750
MAP (mmHg)	108.0 ± 14.8	109.1 ± 16.3	107.6 ± 13.8	107.1 ± 14.3	0.623
RKF (ml/min/1.73m^2^)	6.4 ± 3.5	6.0 ± 2.6	6.4 ± 3.3	6.8 ± 4.4	0.211
Hemoglobin (g/L)	102.2 ± 22.3	101.7 ± 23.3	103.5 ± 23.0	101.4 ± 20.7	0.706
Leukocyte (×10^9^/L)	6.4 (5.2,7.8)	5.6 (4.7,6.8)	6.6 (5.3,8.1)	6.9 (6.0,8.4)	0.002
Neutrophil (×10^9^/L)	4.3 (3.3,5.4)	3.9 (3.0,4.9)	4.5 (1.1,5.6)	4.7 (3.7,6.0)	<0.001
Lymphocyte (×10^9^/L)	1.4 (1.1,1.7)	1.3 (1.0,1.7)	1.3 (1.1,1.6)	1.5 (1.2,1.8)	0.001
Platelet (×10^9^/L)	191.0 (146.0,240.0)	134.0 (111.0,150.0)	193.0 (176.0,213.0)	260.0 (229.0,308.0)	<0.001
NLR	3.0 (2.3,4.1)	2.8 (2.1,3.9)	3.1 (2.6,4.2)	3.1 (2.2,4.5)	0.048
PLR	139.8 (103.3,179.5)	95.5 (76.3,130.3)	147.3 (118.8,174.7)	177.9 (138.1,215.2)	<0.001
Hs-CRP (mg/L)	1.56 (0.62,4.12)	1.06 (0.40,2.77)	1.74 (0.61,5.30)	2.10 (0.93,5.54)	0.007
Albumin (g/L)	35.7 ± 5.6	37.7 ± 5.1	36.5 ± 4.7	32.9 ± 5.8	<0.001
Sodium (mmol/L)	139.6 ± 3.2	140.1 ± 3.0	140.0 ± 2.7	138.6 ± 3.7	<0.001
Potassium (mmol/L)	4.2 ± 0.7	4.3 ± 0.7	4.3 ± 0.7	4.0 ± 0.7	0.002
Chlorine (mmol/L)	103.5 ± 5.4	103.9 ± 5.4	103.9 ± 4.9	102.8 ± 5.7	0.144
Calcium (mmol/L)	2.2 ± 0.2	2.2 ± 0.2	2.2 ± 0.2	2.2 ± 0.2	0.089
Phosphorus (mmol/L)	1.5 ± 0.5	1.5 ± 0.6	1.5 ± 0.4	1.5 ± 0.5	0.744
Parathyroid hormone (ng/mL)	291.3 (156.5,504.5)	320.4 (183.3,487.3)	254.2 (139.7,476.7)	292.6 (161.0,553.8)	0.445
Alkaline phosphatase (U/L)	72.0 (59.0,97.0)	72.0 (59.3,97.0)	71.0 (56.0,88.8)	74.0 (60.0,103.0)	0.108
Uric acid (umol/L)	416.3 ± 99.3	423.9 ± 100.5	418.8 ± 98.4	406.0 ± 98.7	0.317
Triglyceride (mmol/L)	1.6 (1.1,2.0)	1.5 (1.1,2.0)	1.6 (1.1,2.0)	1.6 (1.3,2.2)	0.141
Cholesterol (mmol/L)	4.8 ± 1.1	4.6 ± 1.1	4.8 ± 0.9	5.1 ± 1.2	0.001
LDL-C (mmol/L)	2.8 ± 0.9	2.7 ± 0.9	2.8 ± 0.8	2.9 ± 0.9	0.047
HDL-C (mmol/L)	1.2 ± 0.4	1.2 ± 0.3	1.2 ± 0.4	1.2 ± 0.4	0.603

*p* < 0.05 was considered statistically significant. Values were expressed as mean ± SD, median (25th–75th percentile), or frequency (percent) as appropriate. PAR, platelet-to-albumin ratio; BMI: body mass index; HDL-C: high-density lipoprotein cholesterol; hs-CRP: hypersensitive C-reactive protein; LDL-C: low-density lipoprotein cholesterol; MAP: mean arterial pressure; NLR: neutrophil-to-lymphocyte ratio; PLR: platelet-to-lymphocyte ratio; RKF: residual kidney function.

### Correlation of PAR with the clinical characteristics in PD patients

The correlations of PAR and other clinical laboratory data were shown in Table 2. PAR was positively correlated with platelet (*r* = 0.907, *p* < 0.001), leukocyte (*r* = 0.298, *p* < 0.001), PLR (*r* = 0.614, *p* < 0.001), was negatively correlated with albumin (*r* = −0.364, *p* < 0.001), creatinine (*r* = −0.108, *p* = 0.031) (Table 2).

**Table 2.** Correlations of PAR with laboratory measurements in PD patients.

Variables	Platelet	Albumin	PLR	NLR	Leukocyte	Creatinine	Hs-CRP
r	0.907	−0.364	0.614	0.078	0.298	−0.108	0.159
*P value*	<0.001	<0.001	<0.001	0.117	<0.001	0.031	0.001

*p* < 0.05 was considered statistically significant. PLR, platelet to lymphocyte ratio; NLR, neutrophil to lymphocyte ratio; hs-CRP: hypersensitive C-reactive protein.

### Outcomes of technique failure and mortality

During the median follow-up period of 24.0 months (range: 4.0–91.0), 213 (52.6%) patients continued PD treatment in our center, 13 (3.2%) underwent kidney transplantation, 78 (19.3%) transferred to HD treatment, 23 (5.7%) transferred to other centers, and 17 (4.2%) were lost to follow-up. In total, we recorded 139 (34.3%) technique failure, and 61 (15.1%) all-cause mortality (Table 3).

**Table 3.** Clinical outcomes of PD patients stratified by the PAR.

Variables	Total (*n* = 405)	PAR			*p* Value
		Tertile 1 (<4.51) (*n* = 135)	Tertile 2 (4.51-6.27) (*n* = 135)	Tertile 3 (>6.27) (*n* = 135)	
Follow-data (months)	24.0 (12.0,45.0)	26.0 (11.0,52.0)	21.0 (12.0,37.0)	24.0 (14.0,40.0)	0.267
Technique failure (n, %)	139 (34.3%)	36 (26.7%)	48 (35.6%)	55 (40.7%)	0.048
Death (n, %)	61 (15.1%)	12 (8.9%)	18 (13.3%)	31 (23.0%)	0.004
Kidney transplantation (n, %)	13 (3.2%)	4 (1.5%)	5 (3.7%)	4 (4.4%)	0.924
Other centers (n, %)	23 (5.7%)	8 (5.9%)	9 (6.7%)	6 (4.4%)	0.724
Lost to follow-up (n, %)	17 (4.2%)	5 (3.7%)	7 (5.2%)	5 (3.7%)	0.782

HR: hazard ratio; PAR: platelet-to-albumin ratio. *p* < 0.05 was considered statistically significant.

### Technique survival analysis

The technique failure rates were highest in tertile3 than other two groups (26.7% vs 35.6% vs 40.7%, *p* = 0.048) (Table 3). Kaplan–Meier analyses indicated that the cumulative technique survival rate of patients with tertile 3 was significantly lowest than other two groups (Log-rank = 11.058, *p* = 0.004) (Figure 1). In Cox proportional hazard models, high PAR levels were associated with the risk for technique failure in PD patients (Table 4). In the univariate model, the HRs (95%CI) for the tertile 2 and 3 were 1.791 (1.183, 2.709) and 1.865 (1.244, 2.795), respectively, compared to the tertile 1. In the multivariate model, after adjusting for the confounding factors, including age, gender, BMI, MAP, RKF, comorbidities, NLR, serum sodium, potassium, cholesterol and hs-CRP, the HRs (95%CI) of technique failure for the tertile 2 and 3 were 1.791 (1.183, 2709) and 1.865 (1.244, 2.795), respectively, compared to the tertile 1. When PAR was examined as a continuous variable, the association between PAR and technique failure remained significant (Table 4). The HRs (95%CI) for univariate and multivariate models were 1.147 (1.078, 1.222), 1.148 (1.078, 1.222), respectively. Compared to platelet, albumin and PLR, the HRs of PAR were higher both in univariate model and multivariate model, whether as a categorical variables or continuous variables (*p* < 0.05 for each) (Table 4). This remained the case in the multivariate competing risk analysis with high PAR levels (tertile 3) associated with a higher risk compared to low PAR levels (tertile 1) (SHR 1.775, 95%CI 1.157–2.720, *p* = 0.001) in technique failure and (SHR 3.710, 95%CI 1.870–7.360, *p* < 0.001) (Table 5). The SHRs were still higher than platelet, albumin and PLR, whether as a categorical variables or continuous variables in competing risk regression models (*p* < 0.05 for each).

**Table 4.** Cox proportional hazards models of technique failure and mortality.

Variables	Univariate analysis				Multivariate analysis^a^			
	Technique failure		Mortality		Technique failure		Mortality	
PAR Tertiles	HR (95%CI)	*p* Value	HR (95%CI)	*p* Value	HR (95%CI)	*p* Value	HR (95%CI)	*p* Value
<4.51	Reference		Reference		Reference		Reference	
4.51-6.27	1.791 (1.183,2.709)	0.006	2.073 (0.995,4.319)	0.052	1.791 (1.183,2.709)	0.006	2.248 (1.153,4.382)	0.017
>6.27	1.865 (1.244,2.795)	0.003	3.365 (1.722,6.576)	0.030	1.865 (1.244,2.795)	0.003	3.402 (1.834,6.311)	<0.001
Continuous	1.147 (1.078,1.222)	<0.001	1.253 (1.155,1.359)	<0.001	1.148 (1.078,1.222)	<0.001	1.254 (1.153,1.353)	<0.001
Platelet	1.003 (1.001,1.005)	0.003	1.006 (1.001,1.009)	<0.001	1.003 (1.001,1.005)	0.003	1.006 (1.003,1.009)	0.006
Albumin	0.964 (0.939,0.989)	0.006	0.948 (0.910,0.988)	0.011	0.964 (0.939,0.989)	0.006	0.954 (0.919,0.990)	0.012
PLR	1.002 (1.000,1.002)	0.064	1.004 (1.001,1.006)	0.003	1.002 (1.000,1.004)	0.064	1.008 (1.004,1.012)	<0.001

^a^Adjusted for age, gender, body mass index, mean arterial pressure, residual kidney function, comorbidities, neutrophil to lymphocyte ratio, serum sodium, potassium, cholesterol and hypersensitive C-reactive protein levels. CI: confidence interval; HR: hazard ratio; PAR: platelet-to-albumin ratio; PLR: platelet-to-lymphocyte ratio. *p* < 0.05 was considered statistically significant.

**Table 5.** Multivariate competing risk regression^a^ analysis for technique failure and mortality^b^.

Variables	Technique failure		Mortality	
	SHR (95%CI)	*p* Value	SHR (95%CI)	*p* Value
PAR Tertiles				
<4.51	Reference		Reference	
4.51-6.27	1.598 (1.040,2.460)	0.033	2.164 (1.029,4.550)	0.042
>6.27	1.775 (1.157,2.720)	0.001	3.710 (1.870,7.360)	<0.001
Continuous	1.096 (1.046,1.150)	<0.001	1.173 (1.091,1.261)	<0.001
Platelet	1.003 (1.000,1.001)	0.017	1.006 (1.003,1.010)	<0.001
Albumin	0.965 (0.938,0.992)	0.012	0.954 (0.915,0.995)	0.028
PLR	1.003 (1.001,1.010)	0.004	1.006 (1.004,1.009)	<0.001

^a^Adjusted for age, gender, body mass index, mean arterial pressure, residual kidney function, comorbidities, neutrophil to lymphocyte ratio, serum sodium, potassium, cholesterol and hypersensitive C-reactive protein levels. ^b^Kidney transplantation as a competing event. CI: confidence interval; PAR: platelet-to-albumin ratio; PLR: platelet-to-lymphocyte ratio; SHR: sub-distribution hazards ratio. *p* < 0.05 was considered statistically significant.

### Survival analysis

**Figure 2.**
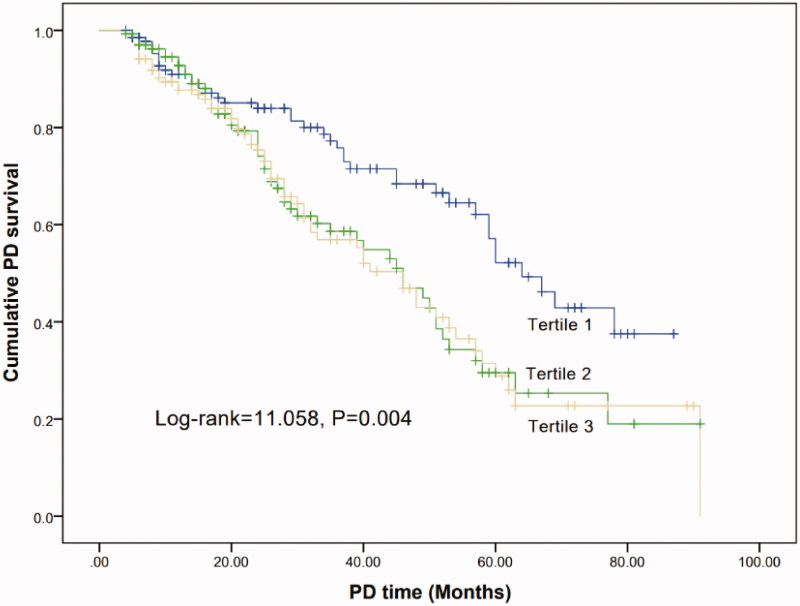
Kaplan–Meier curve of PD survival according to the PAR groups.

The mortality rate was highest in tertile 3 (8.9% vs 13.3% vs 23.0%, *p* = 0.004) (Table 3). Kaplan-Meier analyses indicated that the cumulative patient survival rate of patients with tertile 3 was significantly lowest than other two groups (Log-rank = 14.216, *p* = 0.001) (Figure 2).

The similar trend of technique failure was observed in Cox proportional hazard models, the HRs (95%CI) for the tertile 3 were 3.365 (1.722, 6.576) in univariate model and 3.402 (1.834, 6.311) in multivariate model, respectively, compared to the tertile 1. Compared to platelet, albumin and PLR, the HRs of PAR were higher both in univariate and multivariate models (*p* < 0.05 for each) (Table 4). This remained the case in the competing risk analysis with high PAR levels (tertile 3) associated with a higher risk compared to tertile1 (SHR 3.710, 95%CI 1.870–7.360, *p* < 0.001). The SHRs were still higher than platelet, albumin and PLR, whether as a categorical variables or continuous variables in competing risk regression models (*p* < 0.05 for each). (Table 5).

### Diagnostic value of PAR for technique failure and mortality

**Figure 3.**
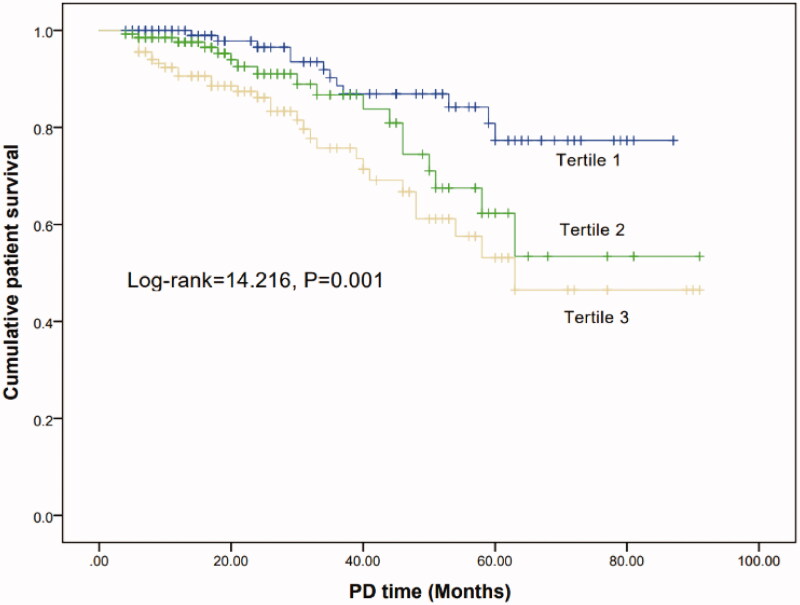
Kaplan–Meier curve of patient survival according to the PAR groups.

The ROC curves were used to compare the predictive power of PAR and platelet, albumin, PLR. The AUC values for the PAR, platelet, albumin and PLR in terms of technique failure and mortality are given in Table 6. Compared with platelet, albumin and PLR, the PAR showed a better predictive power for predicting technique failure and mortality. The optimal cutoff value of PAR was 5.27 for technique failure, with a sensitivity of 59.2% and specificity of 56.2% (*p* = 0.003), and was 5.96 for mortality, with a sensitivity of 60.7% and specificity of 66.0% (*p* = 0.001) (Figure 3).

**Table 6.** AUC using ROC curve analyses to predict technique failure and mortality.

Variables	Technique failure				Mortality			
	AUCs	SE	95%CI	*p* Value	AUCs	SE	95%CI	*p* Value
PAR	0.589	0.029	0.532–0.646	0.003	0.639	0.039	0.563–0.716	0.001
Platelet	0.552	0.029	0.495–0.609	0.079	0.610	0.038	0.535–0.685	0.006
Albumin	0.573	0.030	0.514–0.631	0.014	0.603	0.039	0.526–0.679	0.010
PLR	0.529	0.030	0.471–0.587	0.030	0.594	0.037	0.522–0.667	0.011

AUC: area under the curve; CI: confidence interval; PAR: platelet-to-albumin ratio; PLR: platelet-to-lymphocyte ratio; ROC: receiver operating characteristic. *p* < 0.05 was considered statistically significant.

## Discussion

In the retrospective cohort study of 405 PD patients with a median follow-up of 24 months, we demonstrated that increased PAR levels were significantly associated with higher rate of both technique failure and mortality in PD patients and the PAR was independent predictor for technique failure and mortality. In addition, compared with albumin and platelet, the PAR tended to be a better predictor of poor prognosis in PD patients.

Recent studies have suggested that platelet was positively correlated with various novel inflammatory markers, including C-reactive protein and NLR, and indicated the chronic inflammatory status. Molnar et al. found that higher PLT counts were associated with increased risk of mortality in patients with kidney failure [18]. Peng et al. also demonstrated the association between higher PLT counts and increased risk of CVD mortality in PD patients [7]. In this study, we found that higher platelet counts were significantly associated with higher risk of technique failure, and mortality in PD patients, which was similar to the previous studies.

Low serum albumin levels are considered as a sensitive and classic marker of malnutrition combined with inflammation in PD patients. Considerable previous researches have demonstrated that albumin can indicate poor survival in PD patients. Chen et al. [12] have found that lower serum albumin levels were associated with an increased risk of technique failure. Yu et al. [19] have demonstrated that initial albumin levels were closely related with mortality in PD patients. Similarly, this study also found that low albumin levels were the predictive risk factor for technique failure and mortality after adjusting confounders.

PD patients have always been a clinical condition characterized by the propensity of high inflammatory level and poor nutritional status. Tsai et al. [20] found that the predictive ability of albumin to globulin ratio for mortality risk was superior to albumin in PD patients. Controlling nutritional status score, an inflammation-nutritional index, has indicated to be a reliable prognostic marker of mortality and technique failure in a Chinese retrospective study [21]. In a study of 758 PD patients, serum C-reactive protein to albumin ratio was identified as independent risk factor for all-cause mortality [22]. Increasing researchers preferred to pay more attention to the clinical parameters combining the inflammation and malnutrition in recent years.

The PLT divided by the ALB was regarded as PAR, a new indicator combining with inflammatory status and nutritional status, both of those were tightly associated with PD poor prognosis. Higher PAR, meaning higher PLT counts with inflammation, low albumin levels with poor nutrition, eventually predicting poor clinical outcomes. Previous studies have shown that PAR is an independent predictor in cancer patients, as a clinical marker responding the inflammatory state and nutritional state. Saito et al. [14] have found that preoperative PAR was a prognostic index for disease-free survival and overall survival in patients with pancreatic cancer after pancreatic resection. Guo et al. [15] also demonstrated that preoperative PAR can predict outcome pf patients with non-small-cell lung cancer. However, there are few studies exploring the association between PAR and PD patients. To the best of our knowledge, the comprehensive relationship between the PAR and technique failure in PD patients was revealed for the first time in our study. We found PD patients with higher PAR levels had higher incidence rate of technique failure and mortality. In this study, we showed that PAR was positively correlated with some interrelated prognostic factors for inflammation, including leukocyte and PLR, and negatively correlated with the nutritional factors, including creatinine, suggesting that the PAR may integrate and represent the prognostic value of all of these factors. In multivariable analysis, PAR was the significantly predictive indicator for technique failure and mortality.

In addition, we compared the predictive value of PAR, platelet and albumin. Interestingly, through these three markers were all proven to be independent risk factors for technique failure and mortality, the HRs for PAR were higher than that for both albumin and platelet, and the ROC curve analyses showed that the AUC values for the PAR in terms of technical failure and mortality were largest. Taken together, these indicated that the PAR tended to be a better predictor of poor prognosis than albumin alone and platelet alone.

This study has several limitations. First, this was a single-center retrospective study, and the existence of center-specific effects cannot be completely excluded. Second, we collected baseline PAR only and did not consider the effects of temporal changes in PAR during follow-up. Third, due to the retrospective study, we missed several confounding factors associated with technique failure and mortality, including types of membrane transport, indexes of PD adequacy. The peritoneal equilibration test (PET) is a preferred and frequently used method to evaluate the transport characteristics of the peritoneal membrane, which can decide the optimal treatment regimen for PD patients. Due to the limitations of medical conditions and technological development, we did not routinely carry out PET tests a few years ago. However, previous reports did not support the notion that PET measurements affect the outcome in PD patients. A retrospective study demonstrated that all patients with kidney failure can safely begin standard PD without PET, which only needs to be performed if the patients encounter trouble in total dialysis clearance or fluid removal [23]. Nevertheless, it is a big limitation for the study, and a deep and future study need to gap up the defect. Dialysis adequacy is an important index to evaluate dialysis efficacy and can affect the long-term PD and patient survival, predict the poor prognosis of PD patients. Recent studies have suggested that nutrition parameters (such as albumin levels), and inflammatory parameters (such as CRP levels) should be factored into the assessment of dialysis adequacy. Although the study lacked the traditional indexes of dialysis adequacy, such as Kt/V, the nutritional and inflammatory status have been comprehensively evaluated, which may indirectly reflect the degree of dialysis adequacy of PD patients. Lastly, the possibility of residual confounding could not be eliminated. Multi-site prospective studies are required to confirm these preliminary results.

In conclusion, we proposed a new prognostic index in PD patients. We have confirmed that PAR was a potential risk factor of technique failure and mortality for PD patients.

## Data Availability

The datasets used and analyzed in this study are available from the first author and corresponding author on reasonable request.

## Abbreviations

PD: peritoneal dialysis
PAR: platelet-to-albumin ratio
PLR: platelet-to-lymphocyte ratio
NLR: neutrophil-to-lymphocyte ratio
BMI: body mass index
MAP: mean arterial pressure
CVD: cardiovascular disease
GFR: glomerular filtration rate
